# Housing and health in Israel: the need for local policy-oriented interdisciplinary research

**DOI:** 10.1186/s13584-025-00678-4

**Published:** 2025-03-29

**Authors:** Jordan Hannink Attal, Yehuda Neumark

**Affiliations:** 1 School of Public Health, Faculty of Medicine, Braun School of Public Health and Community Medicine, Hebrew University of Jerusalem, Ein Karem, PO Box 12272, 91120 Jerusalem, Israel; 2 Faculty of Medicine, Melbourne School of Population and Global Health, University of Melbourne, 207 Bouverie Street, Carlton, VIC 3053 Australia

**Keywords:** Housing, Health, Social epidemiology, Housing policy, Israel

## Abstract

**Background:**

Housing is a fundamental condition for health and wellbeing. Housing situation- including affordability, stability, and quality- has been associated with a wide range of health outcomes. Israel is home to a decades-long housing crisis, with housing stock unable to meet demand, lacking housing quality regulation, and few protections for renters.

**Main body:**

This paper presents a review of evidence on housing and health and an overview of the housing situation in Israel. Using a health in all policies framework, we present examples of how public health researchers are leading interdisciplinary research to strengthen the evidence base to change housing policies.

**Conclusion:**

Ultimately, this paper serves as a call to Israeli researchers in the health sciences, urban studies, architecture, public policy, and other relevant fields to take interest in building a local evidence base and promote healthy housing models.

## Background

During the industrial revolution, the foundations of public health were largely based on population-level living and sanitary conditions, including housing. The evident squalor of the proletariat, compared to abundance of the middle and upper class, was a clear determinant of health and wellbeing. In the ensuing centuries and particularly in higher-income countries, public health professionals and public policy experts amalgamized housing and living conditions as part of ‘socioeconomic status’ to capture a myriad factors associated with poorer health outcomes. In doing so, research that substitutes “socioeconomic status” for individual social and economic variables obfuscates pathways between experiencing poverty and poorer health outcomes. As a result, evidence-based social policy interventions may inadequately address the ways in which poverty exposure negatively impacts health, even when using a ‘health in all policies’ approach. While housing has been considered a primary tool of poverty alleviation in many welfare states [1], the quality and stability of housing arrangements and its cultural appropriateness has not been consistently taken into account [2].

Health concerns have certainly changed since the days of the industrial revolution, although the deleterious impact of sub-standard housing on human health and wellbeing remains pertinent. In fact, the UN Sustainable Development Goals (SDG) emphasize access to adequate housing as essential for human wellbeing and sustainable development, particularly in light of the rapidly advancing climate crisis [3]. Indeed, the climate crisis highlights the need for housing that protects humans from extreme temperatures and increasingly intense weather patterns.

In this article, we present an overview of the housing situation in Israel and a justification for why research in the housing-health relationship is of upmost importance for the country’s population health. We then present frameworks for investigating the housing-health relationship, including two examples of countries utilizing surveillance and policy enforcement measurements and two examples of countries employing social epidemiology to influence housing policy. Ultimately, we hope this article demonstrates the dire need for public health professionals in Israel to consider housing as a stand-alone determinant of health.

## Main text

### Frameworks for housing and health

Most high-income countries have building regulations designed to reflect practical, safety, and aesthetic standards. The implementation of such standards may improve health in some settings; however, the beneficial effect of housing standards on health is often inadvertent [4]. Moreover, as sustainable building standards are introduced in the interest of environmental wellbeing, some building standards may be contradictory to elements of healthy housing. For example, requirements for energy saving buildings v. the healthy housing need for adequate ventilation) [5].

Various aspects of housing that may protect or harm human health have been identified.

In 2017, the Health Buildings Program at the Harvard T.H. Chan School of Public Health synthesized multidisciplinary expert opinion into nine foundations for healthy buildings: air quality, thermal health, moisture, dust and pests, safety and security, water quality, noise, light and views, and ventilation [6]. These foundations address all types of buildings, from residential to industrial.

The American Public Health Association’s (APHA) National Healthy Housing Standard shares most of the foundations expressed by Harvard Healthy Building foundations, though they are organized differently [7]. In addition, the APHA adds ‘chemical and radiological agents’ as an additional aspect of healthy housing. This foundation relates exposures to lead-based paint, asbestos, radon, pesticides, and tobacco smoke in multi-residential housing complexes.

Likewise, the National Center for Healthy Housing (NCHH) in the United States shares most of the foundations expressed both by Harvard Healthy Buildings and the APHA Healthy Housing Standard, including “contaminant-free” housing, which includes lead, radon, and asbestos as well as more recently recognized chemical hazards, such as volatile organic compounds and PFAS [8].

The World Health Organization (WHO), in its Housing and Health Guidelines [9], expands on the physical attributes of healthy housing (see Table 1 for full list) to include that “healthy housing provides the feeling of a home, including a sense of belonging, security, and privacy” [9]. Like other frameworks, the WHO extends the housing area to include the immediate environment, which places the housing unit in the context of access to services, green space, active and public transport options, waste management, and pollution.

**Table 1:**
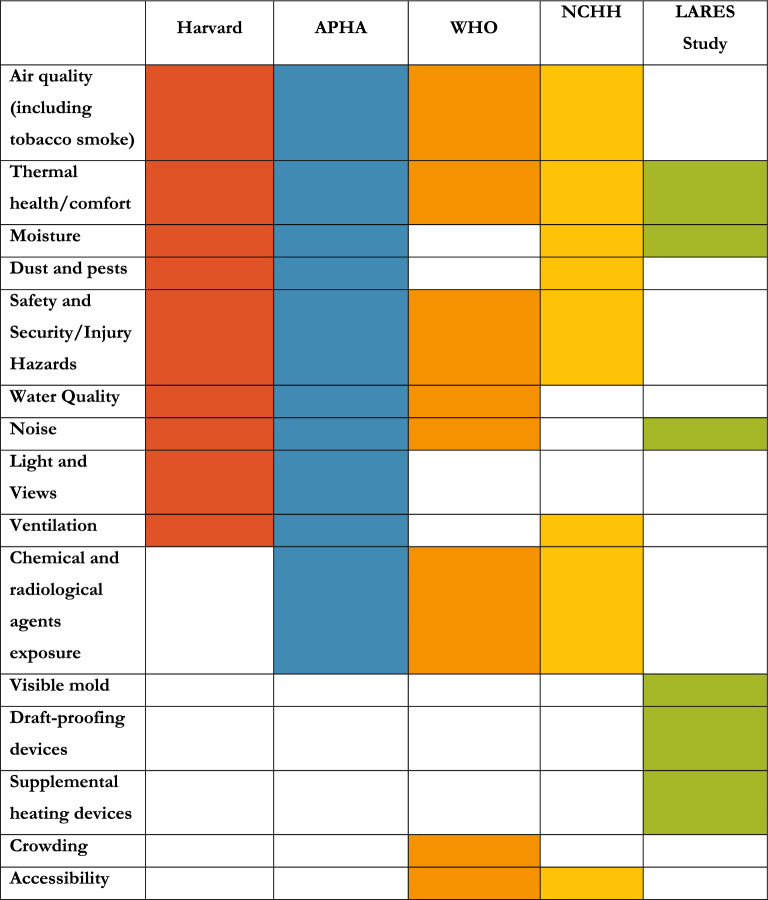
Attributes of Healthy Housing, by Organization

Prior to publication of the WHO Housing and Health Guidelines in 2018, a pan-European survey was conducted concerning housing and health—the Large Analysis and Review of European Housing and Health Status (LARES) [10]. Although the study did not provide a framework for healthy housing standards, we can glean a “framework” from the variables used to determine housing quality. Respondents were asked about perceived thermal comfort, humidity and noise [10]. In addition, surveyors observed the presence of mold, draught-proofing devices, and supplemental heating devices [10].

Most of these frameworks have focused exclusively on potential exposures, yet, researchers have increasingly explored the role of housing affordability, access to social housing schemes, and housing stability as contributors to the housing and health connection [11, 12]. Including affordability and stability parameters in healthy housing frameworks is essential to obtain a more comprehensive understanding of the relationship between housing and health.

## Housing and health in the twenty-first century-evidence from elsewhere

### Housing affordability

Most studies exploring the housing affordability and health relationship focus on mental health outcomes. Overwhelmingly, these studies conducted in multiple countries and political landscapes conclude that unaffordable housing is associated with poorer mental health [13–23].

Housing is considered unaffordable when housing costs account for more than 30% of a household’s disposable income. Many researchers limit this analysis to households in the lowest two income quintiles, earning the measurement the moniker “30/40 indicator”. The relationship between housing affordability and mental health differs by tenure type (whether the home is purchased or rented). Higher proportions of private renters experience unaffordable housing and poorer mental health compared to homeowners with and without mortgages [23–26]. Among homeowners, higher mortgage payments and associated costs were associated with poorer mental health and wellbeing [23–25].

A study in the United Kingdom observed depressive symptoms among private renters receiving and not receiving a housing benefit in a period when the value of the housing benefit was reduced [16]. During the study period, private renters receiving the housing benefit reported a 1.8% (95% Confidence Interval (CI) 1.0–2.7%) increase in depressive symptoms compared to private renters not receiving the housing benefit, pointing to the impact of changes to disposable income on mental health [16]. A comparative study between Australia and the United Kingdom found that when housing became unaffordable, private renters in Australia, but not in the UK, experienced a decline in mental health scores [23]. The authors concluded that the context of rental protections and various forms of welfare support likely mitigate the relationship between unaffordable housing and mental health in the UK for private rentals. These studies indicate the potential for social policy to improve or further damage mental health outcomes among vulnerable populations.

Few studies have examined the housing affordability and health relationship using health indicators other than self-reported mental health surveys [15, 17, 24–26]. Unaffordable rent was associated with putting off seeking medical care for financial reasons among renters in New York City [15], indicating that unaffordable rent is a strong indicator of poor financial health regardless of income bracket. In turn, unaffordable rent may be connected to a variety of health problems (particularly in the United States, which does not have robust universal healthcare) [15]. Another study in the United States (Pennsylvania) found that perceived housing unaffordability was associated with increased poor self-rated health (Adjusted Odds Ratio 1.75, 95% CI 1.33–2.29), hypertension (Adjusted Odds Ratio (AOR): 1.34, 95% CI 1.07–1.69), arthritis (1.92, 95% CI 1.56–2.35), cost-related healthcare non-adherence (AOR 2.94, 95% CI 2.04–4.25), and cost-related prescription non-adherence (AOR 2.68, 95% CI 1.95–3.70) [17]. Likewise, American adults with type-II diabetes were less likely to adhere to medical follow-up when living in insecure housing [26]. A panel study among retirees in the United States that explored the relationship between unaffordable housing and cardiometabolic biomarkers found that retirees with financial strain had higher cardiometabolic biomarker levels, even when adjusting for socioeconomic advantage [24]. Another study using the British Household Panel Study data found that compared with homeowners with mortgages, private renters had significantly higher levels of C-Reactive Protein (CRP), an indicator of inflammation often used as a marker of chronic stress [25]. This finding strengthens the impact of social policy intervention for low-income renters in the UK, as the study also found that housing cost burden was associated with lower levels of CRP for low-income renters [25]. In the United Kingdom, housing cost burden for low-income renters is mitigated by a series of rental protections and a cash-equivalent housing benefit [16, 25].

An additional area of study related to housing affordability is housing stability and instability. Housing stability has been studied in relation to the number of times one has changed their address in a fixed period of time, the receipt of a formal eviction notice, and informal evictions (i.e. landlords taking it upon themselves to change the locks, regardless of legality/illegality). In two longitudinal studies, housing instability was associated with poorer health outcomes, including poorer mental health (whereby each housing move increased the odds of depression by 1.10-fold) and health choices in young adulthood (whereby each additional move increased the odds of regular smoking by 1.12-fold) [27], and generalized anxiety disorder and depression among new mothers (general anxiety disorder AOR 1.9, 95% CI 1.2–3.0; depression AOR 1.4, 05% CI: 1.2–2.3) [28]. Eviction was also found to be associated with depression and poorer self-reported health in studies that examined health outcomes of eviction and forced mobility [29, 30], as well as very low birth weight (adjusted β coefficient: 0.24, 95% CI 0.13–0.34) and higher infant mortality (adjusted β coefficient 1.62; 95% CI 1.11–2.13) [31]. In a longitudinal study of adolescents in the United States, formal and informal eviction during adolescence was found to have both short-term (defined as 12 months) and medium-term (defined as 7–8 years) negative effects on self-reported mental and general physical health metrics compared to those never evicted (short-term AOR 1.05–1.28; p < 0.01) [30].

### Housing quality

In 2011, the WHO’s Regional Office for Europe published a guide which “describes how to estimate the disease burden caused by inadequate housing conditions” [32]. The guide presents methods for modeling the disease burden of a variety of exposures and health outcomes, ranging from well-established and well-described relationships, such as the multiple health effects (including developmental delay in children and cardiovascular disfunction in adults) of lead in housing, to those more difficult to model, such as traffic noise exposure and ischemic heart disease [32]. The diversity of exposures and health outcomes are representative of the multiple, complex pathways between housing and health. While presented separately in the report, many housing quality indicators are interconnected. The potential effect of this interconnectedness is discussed below.

#### Temperature

Studies have shown excess mortality when in-home temperatures are too cold (less than 19 °C) [33, 34]. While studies on the effects of overly warm indoor environments on health are limited, studies suggest too warm (more than 26 °C) indoor temperatures are associated with increased respiratory morbidity, exacerbated mental health disorders, and changes in insulin absorption among diabetics [35]. In Aotearoa New Zealand, a randomized control trial analyzed the health effects of retrofitting homes with insulation, and found reduced odds in occupants’ self-reported poor or fair health (AOR 0.50; 95% CI 0.38–0.68), wheezing (AOR 0.57, 95% CI 0.47–0.70), clinic visits (AOR 0.73; 95% CI 0.62–0.87), days off work (AOR 0.62; 95% CI 0.46–0.83), and children’s days off school (AOR 0.49; 95%CI: 0.31–0.80) [36]. A second study following a policy intervention to retrofit insulation in homes in Aotearoa New Zealand found lower rates of hospital admission rates overall (Rate Ratio (RR): 0.89; 95% CI 0.88–0.90), asthma admissions (RR: 0.80; 95% CI 0.70–0.90), and cardiovascular disease and ischemic heart disease admissions (RR: 0.75, 95% CI 0.66–0.83), particularly among Pasifika peoples of all ages and all people over 65 years [37].

#### Dampness and mold

Inadequately heated and ventilated houses are more likely to be damp, which can result in mold growth [38]. Dampness and mold have been associated with multiple health problems, including asthma development and exacerbation, allergic rhinitis, respiratory infections, depression, and poor overall mental health [39–42]. In addition to people predisposed to asthma, children and women may be more vulnerable to the health effects of mold. A strong, dose-dependent association between visible mold and mold odor and new-onset wheezing was observed among young children in Aotearoa New Zealand (AOR ranged from 1.30–3.56, p ≤ 0.05, wherein a one unit increase in mold exposure equated a 1.46-fold increased odds of wheezing) [43]. The European Community Respiratory Health Survey II, a longitudinal study conducted across 48 cities in 23 European countries, found that dampness and mold exposure were common, and associated with declining spirometry-measured lung function, among women and not men [44]. This finding may reflect a dose-dependent relationship to mold, in which women may spend more time in the home than men.

#### Indoor air quality and ventilation

WHO guidelines point to natural ventilation as essential to reducing the spread of infection [45]. In light of the COVID-19 pandemic, home ventilation in the face of infectious diseases has resurged in relevance [46, 47].

The benefits of natural ventilation are dependent on outdoor air quality. In light of expanding urbanization and the climate crisis, a growing body of evidence has demonstrated that outdoor air pollution influences in-house air quality [48, 49]. Moreover, people in the twenty-first century are spending more time indoors, earning the moniker the “indoor generation” [49]. While there are known associations between air pollution and respiratory diseases, cardiovascular problems, and general morbidity [50], the relationship between indoor air quality and health remains not fully described [49].

#### Household hazards

Household hazards refer to the physical attributes of a residence that may contribute to household injuries (e.g. lack of handrails on stairways, the absence of window guards, or dangerous home fuel storage). According to a 2010 report, nearly 110,000 people die each year as a result of home/leisure injury across Europe, which translates into a fatal injury rate for home/leisure injuries double that of road fatalities [32]. Falls is one of the most prevalent forms of household injury that require medical attention. Modeling studies in Europe noted that falls from windows alone account for 10 deaths annually and 3310 disability-adjusted life years (DALYs) [32]. Household fall risks are particularly acute among vulnerable populations, including people living with disabilities, the elderly, and young children [32]. An intervention initiated nearly five decades ago in New York City to install window guards in households with young children was recently analyzed, and a 91% decrease in falls was observed between 1976 and 2016 [51]. The program became more effective after legal liability for window guards passed from tenants to landlords [51]. Window guards are not the only effective household safety modifications for injury prevention. In a randomized control trial, homes that received household safety modifications, including handrails on outdoor and indoor stairs and grab rails in bathrooms, resulted in a 26% reduction in the fall rate compared to the control group [52].

#### Crowding

Household crowding broadly refers to a situation in which the number of occupants in a home exceeds the space available [53]. The measurement of crowding varies across cultural contexts, and may be measured by the absolute amount of floor space or the number of rooms, and may or may not take into consideration gender, age, and relationship between occupants [9]. Crowding also increases the likelihood of exposure to other housing hazards, such as household injuries [54] and exposure to secondhand smoke [55, 56].

A systematic review included in the WHO Housing and Health Guidelines [9] found that the quality of evidence for a relationship between crowding and respiratory illness, gastroenteritis and diarrhea was high. Previous research has shown the intuitive relationship between pulmonary tuberculosis incidence and household crowding in a variety of settings, including in high-income and low- *mycobacterium tuberculosis* (TB) burden countries [57, 58] In a modern iteration of the relationship between crowding and infectious disease, during the initial COVID-19 wave in New York City the risk of death was two-fold higher in the highest crowding quintile compared to the lowest crowding quintile (RR: 2.58; 95% CI 2.52–2.65) [59].

A different strand of household crowding and health research relates to stress and mental health. While the current quality of evidence is moderate to low [32] due to the types of studies conducted, emerging research suggests a relationship between mental health and crowding [60–64]. For example, among Greenland Inuits, household crowding was associated with higher allostatic load (stress biomarkers) (OR: 2.20; p < 0.001) [63] and poorer self-reported mental health [62]. A panel study from Chile demonstrated that increased household crowding from baseline accompanied more depressive symptoms, whereas consistent household crowding, regardless of degree, was not accompanied by changes in depressive symptoms [64].

#### Holistic measures of housing quality

Some studies have explored housing quality factors more holistically. In a study using the British Household Panel Survey, the persistence of poor housing quality factors–including lack of light, lack of adequate heating, condensation, leaky roof, damp walls, and rot in walls or floor—was accompanied by worsening mental health [65]. The persistence of poor-quality housing was predictive of worse mental health outcomes, irrespective of participants’ current housing quality, which suggests that exposure to poor conditions over time has negative consequences for mental health [65]. Two additional studies on child development, pediatric stress, and housing conditions likewise considered a wider array of housing factors, including crowding, cleanliness, indoor home hazards, and thermal comfort. In both studies, positive relationships between lower housing quality and higher cortisol levels and poorer psychological health were established when using a composite measurement of housing quality [66, 67].

Other studies use both an array of building quality indicators and various health indicators to explore Sick Building Syndrome (SBS) [68], which is a collection of acute health issues that seem to be linked to spending time in a certain building and whose cause cannot be identified nor a particular diagnosis made. Symptoms of SBS include general fatigue, headaches, heavy-headedness, stuffy nose, dry throat, dry eyes, and dry skin [68]. While many studies on SBS focus on industrial buildings, schools, and hospitals, researchers have found a relationship between poor quality home environments and SBS in multiple countries [69–73]. Poor building quality was defined differently across studies, ranging from self-reported sources of indoor and outdoor air pollution, to more robust measurements that accounted for the presence of mold or moldy odor, condensation on windows, and the sensation of too-dry air [71, 73].

#### Sustainable building standards- the health trade-off?

In the era of sustainable building, theoretical literature from the fields of architecture and sustainability research provide support for the potential negative consequences of green building on human health [74–76] For example, in an effort to make buildings more airtight for energy savings, two studies have found lower building air exchange rates and higher concentrations of air pollutants [74, 76]. Similarly, a recent review that examined the impact of sustainable building regulations and indoor mold growth highlighted the incompatibility of sustainable and healthy building regulations [77]. Even if energy-saving buildings are better for maintaining thermal comfort, a green building is not ipso facto a healthy building.

#### Methodological challenges

The absence of robust studies on housing quality and health are largely due to the logistical difficulties of collecting housing quality data. Larger studies that have collected housing quality data chiefly rely on self-report of perceived housing quality issues, such as visible mold or dampness or perceived thermal discomfort. Though these measurements have uncovered associations with multiple health issues, they are not a replacement for more sophisticated and objective measurements, and may underestimate the relationship between poor housing quality and health problems. In addition to the methodological challenge of measuring housing quality exposures, the experience of housing is gendered, whereas women tend spend more time in the home and take on a larger share of household maintenance, regardless of whether or not they engage in paid labor [78, 79]. Additionally, the effects of housing may be more acute for specific vulnerable population groups, including children [31, 54, 67, 80] people with physical and intellectual disabilities [81, 82], and the elderly [83–85] who may spend more time in their home and may be less capable of controlling housing conditions.

## Economizing poor housing

Using a Burden of Disease (BoD) approach allows researchers to evaluate the economic losses caused by poor housing. In the UK, the ‘worst’ housing hazards led to a €1.8 billion expenditure attributable to unhealthy housing [86]. A study of Aotearoa New Zealand on the environmental BoD associated with poor housing (from crowding, cold, damp or mold, and injury hazards) estimated indirect costs of deaths and direct public sector costs at (NZ$) 141 million New Zealand Dollars(NZ$) annually between 2010–2017, and 229 deaths could be annually attributed to poor housing conditions [87]. Considering individual interventions, the cost-benefits analysis of the Housing, Insulation, and Health study found that the health benefits of retrofitting homes with insulation outweighed the costs by nearly two-fold [88]. BoD models are helpful to quantify economic losses and potential gains from improving housing quality, which can shed additional light on how improving housing may reverberate across many quality of life indicators.

## The housing situation in Israel

In the interest of ease of reading, this section is divided into three subsections: housing affordability, housing quality regulations and evidence from the field, and data collection on housing and health in the Israeli context.

### Housing affordability

#### Housing (un)affordability

In the last few decades, the Israeli housing market developed into an infamous ‘bubble,’ with housing costs increasing consistently since the 1990s. Combined with relatively stagnant minimum wage and annual median wages, homeownership remains a distant dream for many Israelis, and rental costs parallel the real estate market. Of all housed people in Israel, over 30% are spending more than one-third of their disposable income on housing alone [89]. The proportion of household monthly earnings spent on housing vastly differs across earning deciles. In 2019, the lowest-earning decile (~ 3000 New Israeli Shekels (NIS) per month/ ~ 940 United States Dollars (USD)) was spending 55% of their disposable income on housing, compared to 20% among those in the highest-earning decile (~ 49,000 NIS/ ~ 15,300 USD) [89]. Given the constant growth in housing costs that outpaces Israeli wages, housing insecurity is expected to increase in the coming decade. This is especially true for women, whose monthly salaries often do not qualify them for a mortgage, nor ensure that they will have affordable housing in the rental market using the 30/40 indicator [90, 91].

Differences in housing quality are found along the lines of an Israeli social dichotomy- the “periphery” and “center.” The periphery is comprised of the northern and southern districts of Israel, which represent 85% of Israel’s land area [92]. Historically, the periphery holds a larger share of public housing units. While the percentage of public housing has decreased, including in development towns in the periphery, the same units that were part of the public housing scheme are now privately owned, and new housing development in the periphery is significantly lagging [92–94].

The center is comprised of the Central, Tel Aviv, and Jerusalem Districts. Though smaller in area, these districts are considered the centers of the economy and government. Jobs, particularly those requiring higher education, are more often located in the center, and as such, average household earnings are higher in the center (except Jerusalem) than the periphery [95]. Together with better jobs and higher earnings, the center has the highest housing demand, and the ability of people living in the center to buy a home/apartment has declined [96].

In light of increasingly unaffordable housing, one solution supported by policy makers is encouraging residents to relocate to the periphery, and offering tax benefits for doing so. While housing prices are more affordable in the periphery than the country’s center, buildings are more likely to be older and of poorer quality than those in the center [97]. Moreover, while urban regeneration projects are touted as the solution to poor and dangerous buildings, none of these projects exist in the periphery. In 2022, 12 municipalities entered or extended framework agreements for urban regeneration- all of which are centrally located. Though a recently adopted plan allocated funding for urban regeneration in the periphery, the benefits for building improvement given to apartment-owners in the periphery are less than those given to apartment-owners in the center. For example, whereas apartment-owners in the center receive an additional room and balcony as benefits for agreeing to urban regeneration projects, apartment owners in the Periphery are not entitled to these benefits [98]. In addition, as an effort to increase building safety in the event of an earthquake or missile bombardment, a second urban regeneration plan allows developers to strengthen existing buildings while adding additional apartment units. A recent Knesset Research Information Center report found of the 70 buildings that have been reinforced in the past five years, only 5% were located in the periphery [99].

While buildings in urban regeneration frameworks are constructed using the latest building codes, the sustainability and durability of new construction will depend on the quality of materials, craftsmanship, and rigor of building codes and their oversight. According to a report commissioned by MoCH in 2010 (the last time such a report was published), 100% of new build apartments had construction defects, most of which are attributed to an unskilled workforce and lack of effective oversight [100]. Notably, some of these defects are not readily reparable after construction- particularly defects in sealant and piping, which contribute to housing quality issues for decades [100]. While developers and contractors are required to extend warranties to buyers, these warranties are not always honored [101, 102]. Buyers have the option to sue to developers or contractors, and indeed 91% of lawsuits against developers for construction defects awarded the plaintiffs, however lawsuits are uncommon relative to the frequency of defects [101]. Related to housing unaffordability, the costs of defects are included in market price- contributing 10% of the overall cost of the apartment in 2014 [102].

#### Affordable housing programs in Israel

Israel’s housing affordability policies can be divided into two overarching programs—one called “Apartments at Discount” which aims to assist first-time buyers, and Housing Assistance in the form of social housing or rent assistance stipends.

In an effort to make housing more affordable for first-time buyers, the Ministry of Construction and Housing (MoCH) offers four affordable housing tracks (described in Table 2), collectively referred to as “Apartments at Discount” (AaD). All Israeli couples, singles over the age of 35, or people with a high level of disability and over the age of 21, and who have not owned a property in the last 5 years, are eligible for the AaD program, though some differences between the tracks exist. After applying to the program, selection is based on a lottery system per project. Apartments purchased through these programs cannot be sold for 5 years from receipt of the occupancy permit, or 7 years from the time of the lottery, whichever comes first.

**Table 2:** State of Israel Ministry of Construction and Housing’s Affordable Housing Program Tracks (‘apartment at discount’ tracks), 2024

Name of track	Details
Goal price (*Mechir Matera*)	The discount given is 20% of the listed price (including tax) or 300,000 NIS (the lower between the two), and the price per square meter is limited to 20,000 NIS. Projects in this program must be located in municipalities in the 1st-4th Socioeconomic Clusters (defined by CBS)
Reduced price (*Mechir Mufehat*)	This program was implemented in 2020–2021, and the lottery has yet to be drawn for these projects. In exchange for discounted land, developers competed in a tender process to provide the lowest possible price per meter squared, without limitations on what that price could be (to differentiate from mechir l’matera). In addition, participants selected through the lottery system receive an additional 40,000 NIS
Buyer’s price *(Mechir l’Mishtaken)*	Started in 2015, this program auctions land to developers at a significantly reduced price who can also answer to a tender to provide low-cost housing. Preference is given to those who are staying in a municipality where they have lived in the recent past, with the exception of high-demand areas. Up to 90% of the apartment can be financed (compared to 75% on the private market) and require a minimum down payment of 100,000 NIS. In addition, special grants for people relocating from the center to the periphery are available, ranging from 40,000–60,000 NIS
Young buyer (*Mishtaken Tzair*)	For single people (including divorcés and widows) between 26–34 years old, apartments that remain after lottery drawings are available to young singles for purchase with the same discounts offered through the AaD track. Selection of mishtaken tzair applicants is not based on a lottery system

From 2015 to August 2018, 128,100 eligible households applied to the various AaD tracks. Of the 64,000 units that were approved for construction, only 11,780 units received building permits [103]. According to a 2020 report from the State of Israel Comptroller’s Office, one of the AaD tracks (“Buyer’s Price”) had several shortcomings, including not accounting for applicants’ needs, lack of availability of smaller apartments, delayed timelines, and failure to limit participants to their existing residential areas (which leads to the units being rented out upon completion, rather than owner-occupancy) [103].

In addition to AaD, people who meet eligibility criteria also qualify for a reduced long-term rental program called “Apartment for Rent”. This program, operated by a government-owned company, provides long-term rental properties 20% below market rental value for a period of 10 years. Participants are selected through a lottery system, similar to AaD. Despite its implementation in 2013, only 10,000 apartments were made available through this program as of June 2022 [104].

Both the AaD and Apartment for Rent programs offer selected applicants the possibility for stable long-term housing arrangements at attractive prices. Given the volatility of the Israeli housing market [97], selection for such programs may provide the selected applicants with a greater sense of control over their lives compared to those in the private market.

Additionally, given that units provided through AaD and Apartment for Rent are new builds under strict supervision from state-employed regulators, the quality of apartments through these programs may be better than what is available and affordable to applicants on the private market.

#### Social housing programs in Israel

Today, only 2.5% of the national housing stock is public housing, placing Israel among the lowest-ranking countries in the OECD in terms of public housing [105]. It is noteworthy that in the first decades of statehood, about 60% of all housing was public [89]. In absolute terms, between 2014 to 2020, the quantity of housing supply decreased from 60,000 units to 53,000 units, or a decline of 2% annually on average [105]. In 2018, the government launched the “To live with respect! Saving Public Housing” campaign, which committed to acquiring a minimum 1000 units per year and totaling 73,000 units by 2028. Yet, only 234 units were acquired between 2014–2020 [105].

The demand for public housing is not caused by overly inclusive eligibility criteria for receiving public housing. In fact, the length of the waiting list was stabilized in the last two decades by tightening eligibility criteria. First, a household must be considered houseless, legally translating to not owning an apartment or a share of an apartment in the last ten years, nor having received financial compensation for housing, except rental assistance, or protected housing except public housing since 1971 [106]. Second, households must qualify for income supplements through the National Insurance Institute (*Bituach Leumi*) for a minimum of 24 months [106]. Third, households requesting public housing must belong to one of the following categories:

- Elderly (> 75 years of age).
- Single people with a permanent disability of > 75% that prevents them from supporting themselves.
- Families, including single-parent families, with 3 or more children.
- Families, including single-parent families, with a dependent child with 100% disability.
- Married couples in which one spouse has a disability of > 75% with 2 or more children.
- Married couples in which each spouse has > 75% disability, with at least one child (not including pregnancy).
- Immigrants with Israeli citizenship unable to secure housing (up to 15 years after their immigration date).

Despite these stringent eligibility criteria, there are currently over 30,000 households waiting for public housing [92]. In part due to the eligibility criteria, women are overrepresented, accounting for 62% of public housing residents [90]. Among women in public housing, 75% were either single-mothers or single [90]. Despite this high proportion of single-parent households in public housing, many single-mother households are not eligible for public housing.

The second available route to housing assistance is rental assistance through the MoCH. In recent years, rental assistance has become the housing assistance route preferred by the Ministry of Finance (MoF), whose position is to sell all public housing holdings and shift solely to rental assistance [105, 107]. While the number of households receiving rental assistance stood at 138,000 in 2010, shifts in policy that prefer rental assistance led to a significant increase of recipients over the last decade, and there are currently over 180,000 households receiving rental assistance [108]. Despite the preference of the MoF to turn households in need of assistance to the free market, the amount of rental assistance has not financially liberated these households. The proportion of average rental assistance to average rental costs in the free-market decreased from nearly 30% of average rent in 2000, to 24% in 2020 [108]. The decrease in this proportion is largely due the absolute amount of rental assistance remaining stagnant since 2002 [109]. For Israelis who receive rental assistance through the Ministry of Immigration and Absorption, rental assistance is expected to decrease in absolute value in 2024 due to budget cuts, amounting to a per household reduction of 100–400 NIS per month [110].

Similarly to the qualifications for public housing, to qualify for rental assistance households must meet the definition of “without housing” and earn at least minimum wage [106]. The average monthly rental assistance is 860 NIS (~ 270 USD). However, households receiving rental assistance are often met with stigma and sometimes experience problems securing housing in the private market [111–113], and may face discrimination in securing other welfare benefits (such as a reduction in municipal tax) [114]. The combination of stigma of people receiving rental assistance and lack of oversight on the private rental market may lead to the exploitation of people who receive rental assistance.

### Housing quality regulations and evidence from the field

#### Housing quality regulations

Housing policy in Israel offers little in terms of regulation for housing quality. The legal definition for an inhabitable space is based on five parameters: 1) the space has ventilation and natural lighting; 2) the ability to close and lock all entrances to the residence; 3) the space has working water and plumbing infrastructure; 4) there is a partition between the bathroom and the living space; 5) the space has an electrical and lighting system. While a sixth parameter is listed by law that the space “does not pose an unreasonable risk to tenant’s safety or health,” no legal definition for “unreasonable risk” is offered [115]. The minimal definition of habitability translates into housing quality that depends solely on the sensibilities of developers, homeowners or landlords. This is also true in the case of public housing, where state-owned companies manage properties. In 2021 alone, as a result of “cost-cutting measures” in apartment maintenance, the largest state-owned public housing managing company (*Amidar*) profited by 150% (370 million NIS; 115 million USD) compared to the previous year’s earnings [116]. According to the State Comptroller’s annual report, 72% of public housing units were in need of repairs, of which 25% required serious renovation [117]. The primary housing condition issues described in reports spanning decades have included mold, lack of functional plumbing and electrical infrastructure, water damage, and cracked walls and foundation [92, 105].

### Sheltered areas- a uniquely Israeli housing quality parameter

Israel’s security situation—namely, being surrounded by two hostile states and the Gaza Strip, necessitates protection measures now rare in high-income countries in Western Europe, North America, and Australia. All residents of Israel are to have access to a bomb shelter. Israeli law recognizes three types of bomb shelters- apartment safe rooms (MaMaD in Hebrew), floor safe rooms (MaMaK), and institutional bomb shelters (MaMaM). In addition, each municipality is responsible for public bomb shelters, although in 2019, 28% of public shelters were found to be unfit for use, and another 27% were in a poor state [118, 119].

In 2020, 60% of residential units did not have apartment safe rooms, and 27% of residents did not have access to a sheltered area close to their home [119]. The government plan to improve shelter access defined prioritized areas within 40 km of hostile borders, and further divided these 40 km into four distance categories. At the time of last analysis, 15% of residents within four kilometers of hostile borders, and 30% of those within 5–40 km, did not have access to an adequate shelter [120]. Border towns in the Gazan Envelope have higher rates of shelter access due to government prioritization [120]. However, Northern-border communities have yet to benefit from the shelter improvement law, largely due to government cutbacks in 2022—from 750 million NIS over two years to 100 million NIS over the same time span [120].

In 1991, a law was passed that required all new residential buildings to include a safe room in each unit or on each floor [121]. This includes buildings undergoing Urban Renewal projects (TAMA-38). In 2016, when TAMA-38 was renewed, in-unit shelters were mandated in the updated regulations [122]. As noted above, TAMA-38 projects have nearly exclusively focused on municipalities in the Center, where profit margins are considerably higher than in the Periphery. Without government programs and without market interests for developers, this leaves many border-town residents to finance their own shelters. The average cost to construct an apartment shelter is 140,000 NIS ~ 43,000 USD) [123].

### Household density

In a preceding paragraph in this article, we addressed that crowding measurements should take into account shared space, room size, relationships between household members, and their gender. The CBS calculation for household density divides the number of household members by the number of rooms [124]. The CBS measurement does not take into account the relationships between household members and their demographics are not taken into account and the number of rooms includes shared living spaces, augmenting available space.

Nevertheless, this data provides general insights into housing density in Israel. According to data collected in the 2023 Social Survey, 50.3% of Israeli households had a housing density of 0–1 persons per room, 42.1% had 1–2 persons per room, and 6.6% had more than 2 people per room, with no discernable relationship with net household income (see Table 3) [124]. When examining housing density and tenure, homeowners report lower density (0–1 persons per room: 53.7%) compared to renters (0–1: 44.8%) (See Table 4). Some important trends begin to be distinguishable when considering sect and homeownership (See Table 5) [124]. Compared to non-ultra-Orthodox Jews, ultra-Orthodox (Haredi) Jews and Arabs had lower percentages of 0–1 persons per room, with the highest distribution category being 1–2 persons per room. Whereas non-Haredi Jews did not report having 2 or more persons per room, 21% of Haredi Jews and 16% of Arabs reported having 2 or more persons per room [124]. These distributions differ by tenure type—higher density accompanies homeownership, which may indicate a trade-off, in which ownership stability is prioritized over apartment size.

**Table 3:** Housing density by net household income, CBS Social Survey 2023

Net household income	Household density (people per room)		
	0–1	1–2	2 +
< 4000 NIS	110,753	82,628	
%	57.3%	42.7%	
4001–6500 NIS	225,375	149,010	52,760
%	52.8%	34.9%	12.4%
6501–10,000 NIS	334,299	313,718	102,956
%	44.5%	41.8%	13.7%
10,001–13,000 NIS	224,215	220,274	56,911
%	44.7%	43.9%	11.4%
13,001–17,000 NIS	337,337	334,769	44,826
%	47.1%	46.7%	6.3%
17,001–24,000 NIS	398,190	379,980	28,220
%	49.4%	47.1%	3.5%
> 24,000 NIS	446,561	342,746	
%	56.6%	43.4%	
Unreported	653,614	608,685	75,518
%	48.9%	45.5%	5.6%
Total	2,730,344	2,431,810	361,191
%	49.4%	44.0%	6.5%

**Table 4:** Housing density by tenure, CBS Social Survey 2023

Tenure type	Housing density (persons per room)			
	0–1	1–2	2 +	Total
Homeowners	2,201,408	1,636,938	232,419	4,070,765
**%**	54.1%	40.2%	5.7%	
Renters	751,727	784,949	131,913	1,668,589
**%**	45.1%	47.0%	7.9%

**Table 5:** Housing density by sector and housing tenure, CBS Social Survey 2023

	Housing density (persons per room)			
	0–1	1–2	2 +	Total
Ultra-orthodox (Haredim)	25%	51%	21%	
Homeowners	20%	31%	14%	65%
Renters	5%	20%	7%	33%
Jewish- not ultra orthodox	59%	34%	0%	
Homeowners	44%	22%		67%
Renters	15%	12%		28%
Arab	20%	50%	16%	
Homeowners	17%	44%	11%	73%
Renters	3%	6%	5%	14%
Total	48%	39%	6%	
Homeowners	36%	26%	4%	66%
Renters	12%	13%	2%	27%

### Special populations: the Haredi sector

The Haredi sector is the fastest growing sector in Israeli society, projected to grow to ~ 20% by 2035 [125]. Haredi society functions as a collective, and Haredi Jews have a strong preference, if not requirement, to live in a geographical area with necessary religious infrastructure. To address the growing demands to this specific population, MoCH compiled a strategic plan for housing in the Haredi sector [126]. This plan includes the addition of 200,000 housing units between 2016–2035, averaging 10,000 additional units per year. The additional units are to address half of the expected demand in the Haredi sector [125]. However, the annual targets have not been met in any year since the plan’s adoption, and the approval rate for new construction in existing Haredi neighborhoods is lower than in non-Haredi neighborhoods [125].

As mentioned in the previous section, the Haredi sector has higher housing density compared to non-Haredi Jews. The strategic plan primarily addresses the availability of housing units, rather than their size or features.

### Special populations: Israeli Bedouins residing in recognized and unrecognized villages

The State of Israel is home to more than 300,000 Bedouins—3.3% of Israel’s total population. Most of Israel’s Bedouin population resides in the Negev Desert, of whom more than 70% reside in recognized villages [127]. Under the Planning and Building Law [128], structures built in unrecognized villages are illegal, and state authorities are not obligated to provide basic infrastructure to these villages, including connection to national electrical grids, water and sewage systems, and/or paved roads [128]. Given that building in unrecognized villages is illegal, these villages are largely comprised of temporary dwellings [129].

In one of the only studies concerning housing and health in Israel, the threat of home demolition was associated with depressive symptoms among Bedouin women [130].

Bedouins residing both in recognized and unrecognized villages have a shortage—at best— of sheltered areas, despite living within 40 km of the Gazan border [118, 131]. Furthermore, the Iron Dome air defense system does not protect unrecognized villages from rockets fired into Israel [131]. Despite a ruling in 2014 that temporary bomb shelters must be provided to residents in recognized and unrecognized villages, at the time of this writing, this plan has not manifested meaningful results [131].

### Data collection on housing and health in Israel

Collection of housing indicators data in Israel, both in terms of affordability and quality, is lacking. The only housing indicators included in the national decennial census (conducted by the Central Bureau of Statistics—CBS) are tenure type, monthly housing payments, number of rooms, and household size. The latter two indicators are used to calculate household crowdedness (rooms per person). Some financial indicators of housing, such as monthly payments, are also tracked by the Bank of Israel.

The census does not collect health information, which precludes using census data to assess housing-health associations. While one could theoretically cross-reference housing data collected by CBS with personal health data from the health maintenance organizations, it is questionable if such analyses would yield meaningful results given the limited scope of available data and inability of follow-up due to the census methodology.

## Envisioning the alternative: international examples of public health researchers building a policy oriented evidence base

In this section, we present two country cases for surveying and enforcing housing standards- the United States and England and Wales, and two country cases in which healthy housing research frameworks have, and continue to translate into policy changes—Aotearoa New Zealand and Australia. While some similarities exist, the progression of policy in these two cases are quite different. These country cases can serve as models for building an Israeli healthy housing research framework in practical terms.

### United States

Tracking the prevalence of potential housing related health hazards is important to justify both research on the housing-health relationship and ongoing policy development. The evidence for the power of surveillance in leading to quantifiable change is the US experience of policies aimed at reducing household lead exposure, particularly among children, since its recognition as a hazard in 1971 [132]. Surveys that measure the ongoing potential for exposure to known household hazards identified by the Environmental Protection Agency are conducted by the US Department of Housing and Urban Development’s (HUD). In the last 25 years, HUD has measured housing hazards in three surveys: the National Survey of Lead and Allergens in Housing (1998–9), the American Healthy Homes Survey (2005–6), and the American Healthy Homes Survey II (2018–9). The most recent of these surveys, the American Healthy Homes Survey II, measured levels of lead, lead hazards, pesticides, formaldehyde, mold, moisture damage, smoke alarms, carbon monoxide detectors, fire extinguishers, smoking, pests, electrical hazards, hot water availability, and slip hazards. in a nationwide study including over 700 homes where children may live [132, 133]. This survey found that lower income and owner-occupancy were associated with a higher frequency of housing hazards, as were older homes. Regional differences were also noted [133].

It is important to note that while HUD has invested in surveying the prevalence of hazards and many organizations in the United States have created healthy housing guidelines, little research in the United States has focused on the relationship between housing and health in the last two decades. Such surveys are insightful for researchers and policy makers in estimating the prevalence of housing hazards already deemed pathogenic for certain health outcomes, although they do not offer the ability to measure the effects of persistent exposure to these hazards or to determine the relationship between these recognized hazards and other pathologies. Even with known hazards, such as lead, surveillance data for housing exposures and health outcomes are collected by different agencies on different timelines, making epidemiological modeling particularly difficult and leading to limited renewed policy oversight [134].

As highlighted above, private renters are particularly vulnerable to substandard and unaffordable housing in many developed economies, including the United States. Addressing potential hazards in rental housing requires knowing which housing is used as rental housing. Rental registries are recent policy tool for state and local governments in surveying and enforcing standards in private rentals. Rental registries are formal registries kept by local governments that monitor and regulate rental housing, typically collecting data on the landlord and their holdings, safety and building code compliance, and tenant protection enforcement [135]. Some registries require unit inspections to ensure compliance with local building codes. Some states or cities, including New York, California, and Portland, Oregon require rental registry for certain types of properties, namely properties that fall under rent control protections [135–138].

While the range of rental registry procedures and oversight currently drastically vary, the oversight provided by the act of registry may be an important step in improving housing hazards for private renters in the United States. However, building codes are not synonymous with healthy housing standards, even when registries require inspection to ensure rental units meet building codes,

### England and Wales

England and Wales have a long history of slum clearance for public hygiene and housing standard legislation [139–141]. Historically, enforcement mechanisms have been central to healthy housing standards in England and Wales, including in their modern iterations. In 2004, the Housing Health and Safety Rating System (HHSRS) was introduced as part of the Housing Act, and has been enforced since April 2006 in England and Wales [142]. The HHSRS is the main system for assessing and enforcing housing standards on the basis of potential risk to health and safety for tenants. While the HHSRS does not discriminate between tenures (private ownership, private rental, or social rental), the HHSRS addresses an acute need in the private rental market, where tenants are the most vulnerable to poorly maintained housing [143].

In essence, a tenant who believes their housing to be unsafe can file a claim through their local council and request an HHSRS assessment, which is performed by an environmental health officer. The environmental health officer and the council are responsible for informing the landlord of the results of the assessment, and actions that should be taken to ensure safety and adherence to standards. The range of actions taken depends on the severity of the violation, and failure to address unsafe housing can result in punitive action against the landlord, including civil penalties (up to 30,000 Pound Sterling), a banning order forbidding a landlord from renting for a period of time, and rent restitution [142].

The HHSRS has been reviewed several times since its initial adoption, namely for lacking clarity for landlords and tenants as well as lacking enforcement for comfort standards (as opposed to safety standards). As the result of a review of the HHSRS in 2015, standards were unchanged, but a comprehensive guide for renters was published, which includes a description of the standards and steps to take to enforce standards. For property agents and landlords, two additional checklists were published to facilitate compliance with the HHSRS standards. After the 2017 Grenfell Tower fire, additional standards for cladding in high-rise buildings were added to the HHSRS standards, allowing for a consideration of shared building conditions rather than solely individual units [142].

In 2019, the Homes (Fitness for Human Habitation) bill, secondary to the 2004 Housing act, was passed, and places the legal responsibility on landlords (both private and corporate) to ensure housing meets property standards before and during tenancy in England [144]. Simultaneously, the bill creates legal pathways for tenants to pursue legal action against their landlords for failing to address substandard properties [144]. The bill also protects tenants from retaliatory eviction by forbidding landlords from serving eviction notices within six months of filing a claim concerning substandard conditions [144].

### Aotearoa New Zealand

Aotearoa New Zealand is home to the He Kāinga Oranga- Housing & Health Research Programme and WHO Collaborating Centre on Housing and Wellbeing, based at the University of Otago, Wellington. The program is operated in partnership with the New Zealand Centre for Sustainable Cities, Māori, local government, and the community and funded by the Health Research Council of New Zealand and the Ministry of Business, Innovation, and Employment [145]. The Research Programme was founded in 2001, with the overarching research goal “to contribute to the policy evidence needed to improve the quality and supply of sustainable housing, and hence health and wellbeing” [2]. The multidisciplinary team uses a community-based participatory approach to conduct their research, and interventional studies are a key feature of their research methodology and community obligation [2].

Research conducted in the framework of this program is having a direct impact on housing policy. For example, findings from research concerning indoor temperature and reduction in health harms catalyzed the introduction of the Warm Up NZ Programme in 2009, a government program which co-funds the retrofitting of insulation and clean heating sources [146]. When evaluated for costs, the program was found to have sizeable net benefits. Most recently, the totality of the He Kāinga Oranga- Housing & Health Research Programme’s research contributed to the adoption of the Residential Tenancies (Healthy Homes Standards) Regulations by New Zealand’s Ministry of Housing and Development in 2019. These regulations set the minimum standards for heating, insulation, ventilation, dampness and drainage, and draught-stopping for rental properties [147].

### Australia

Housing and health policy-oriented research in Australia is conducted by two organizations, one tangentially- the Australian Housing and Urban Research Institute (AHURI)- and the second exclusively- the Healthy Housing NHMRC Centre for Research Excellence.

AHURI was founded in 1993 as a joint research center between academic and policy players to address issues of housing affordability, sustainable cities, and built environments [148]. AHURI’s scope and funding expanded in 1999, and it continues to operate as a state-funded research hub on housing issues in key policy areas, including housing systems, social and affordable housing, planning and urban policy, indigenous housing, homelessness, the private rental sector, home ownership, and housing vulnerable people. Their research is compiled into special reports with key policy recommendations [148].

In 2022, AHURI sponsored and published a report titled “Precarious housing and wellbeing: a multi-dimensional investigation” [11]. Based on the study’s key findings, the research team suggested three themes in addressing the most immediate needs of people in precarious housing, including changes to regulations in providing housing assistance to the most vulnerable, regulation of private rentals, and the integration of housing and non-housing policy measures for vulnerable groups, including young people entering the low-wage labor force and people at risk for homelessness [11].

In 2020, the Australian government’s National Health and Medical Research Council (NHMRC) designated funding for a research group on Healthy Housing, in partnership with Australian universities, University of Otago, the National Center for Healthy Housing in the United States, and the South Australian Medical Research institute. The Healthy Housing NHMRC Centre for Research Excellence (Healthy Housing Centre) objectives include “transfer[ing] research outcomes into health policy and decision making in policy and practice at the state and federal levels in Australia, and internationally via the WHO Healthy Housing platform” and “develop[ing] Australian research capacity in the interdisciplinary sectors of housing, health, and policy implementation” [149]. The Healthy Housing Centre focuses on three ‘streams’ of research: how housing affects health over time; health gains and losses from housing; and capturing complexity in the housing system. Each stream is composed of several complimentary research projects.

In addition to other data sources, the Healthy Housing Centre has benefited from data collected in Household, Income, and Labour Dynamics in Australia panel study (HILDA). HILDA, funded by the Australian Government Department of Social Services and managed by the Melbourne Institute of Applied Economic and Social Research at the University of Melbourne, has regularly collected data on health (Standard Form-36), housing affordability and tenure, as well as other parameters in a representative sample of Australians [150].

In its short tenure, the Healthy Housing Centre has waded into housing affordability and supply policy in Australia, and has submitted a Submission to Inquiry to the Australian Parliament [151]. During the initial COVID-19 waves, the Healthy Housing Centre worked to inform policy makers of unintended consequences of lockdown measures on housing precarity by creating the Neighbourhood Employment and Housing Precarity Index, mapping neighborhoods in the major urban hubs of Melbourne and Sydney [152]. A forthcoming inquiry involves the readiness of Australia’s housing stock to withstand the effects of the climate crisis, following flooding and extreme temperatures in 2023 [153].

## Next steps for building evidence-based policy for healthy housing in Israel

Israel stands at a crossroads in housing construction, given the demographic pressure to build additional units as well as geological and political pressure to improve building safety in terms of earthquakes and rocket bombardment. The impetus to build renders the lack of comprehensive housing policy in Israel—including the regulation of quality, effective housing affordability programs, renter protections, and adequate housing stock- impermissible. No longer can housing planning be siloed into the realm of market interest or the laissez-faire approach of the Ministry of Housing and Construction. As demonstrated in studies described in-depth above, housing has direct effects on health, including in countries with stronger housing regulations and social housing programs than Israel.

In order to inform local policy actions, a multidisciplinary research working group or center, similar to those in Aotearoa New Zealand and Australia, should be established, and ideally funded by the state. This could be accomplished through inter-ministerial funding and partnership with local universities and research centers. Building on strong causal evidence from elsewhere, the immediate objective of such a research group should include an assessment of the local magnitude of housing problems that contribute to poor health outcomes. A second primary objective should include an assessment of Israel-specific housing factors, such as the presence and adequacy of sheltered areas, the efficacy of existing public housing and housing benefits, and the effect of TAMA and AaD programs in regards to health and wellbeing.

Internationally, much of the current research on housing and health outcomes is achievable due to regularly collected panel data which includes both housing and health indicators in established representative cohorts. These data are collected both by government agencies (such as the HILDA survey) and by research universities (such as the Future of Families and Child Wellbeing Study of Princeton University and Columbia University [154]. In Israel, data concerning housing affordability, diagnosed health problems, and household crowding is held by different national agencies. Inter-agency partnerships should be promoted, and data linkages and collection should be encouraged and funded to create a national housing-health data platform.

Given the plurality of minority and vulnerable subpopulations in Israel, special attention should be given to adequate sample sizes in subpopulations and community-based methods should be used when appropriate [2]. Additionally, while it is acknowledged that the experience of housing is gendered, few studies have employed feminist analysis in exploring the housing and health connection [155]. Evidence from Israel regarding the gendered experience of housing affordability and the overrepresentation of women in public housing [90] provide a particular impetus for trailblazing feminist and gender-sensitive approaches to housing and health research.

While initially tasked with providing an evidence-base for policy change, the long-term vision of such a research group should include monitoring changes on the ground following policy intervention.

## Conclusion

Improving housing is a clear step to increase social equity for the betterment of the socioeconomically disadvantaged. Evidence-based policy interventions in housing stock, quality regulation, and housing welfare programs are not solely of national economic interest, but a moral imperative in a democratic government and society.

## Data Availability

Not applicable.

## Abbreviations

AaD: Apartment at discount
AHURI: Australian housing and urban research institute
AOR: Adjusted odds ratio
APHA: American public health association
BoD: Burden of disease
CBS: Central Bureau of statistics
CI: Confidence interval
DALYs: Disability adjusted life years
HHSRS: Housing health and safety rating system
HILDA: Household, income, and labour dynamics in Australia
HUD: US Department of housing and urban development
LARES: Large analysis and review of European housing and health status
MoCH: Ministry of construction and housing
MoF: Ministry of Finance
NCHH: National center for healthy housing
NHMRC: National health and medical research council (Australia)
NIS: New Israeli Shekel
NZ: New Zealand
RR: Rate ratio
SBS: Sick building syndrome
SDG: Sustainable development goals
TB: mycobacterium tuberculosis
USD: United States dollars
WHO: World Health Organization
